# Multidimensional primate niche space sheds light on interspecific competition in primate evolution

**DOI:** 10.1038/s42003-024-06324-0

**Published:** 2024-05-27

**Authors:** L. A. van Holstein, H. D. McKay, C. Pimiento, K. Koops

**Affiliations:** 1 Clare College, University of Cambridge, Cambridge, UK; 2 Ape Behaviour & Ecology Group, Department of Evolutionary Anthropology, University of Zurich, Zurich, Switzerland; 3 Department of Archaeology, University of Cambridge, Cambridge, UK; 4 Department of Paleontology, University of Zurich, Zurich, Switzerland; 5 Department of Biosciences, Swansea University, Swansea, UK

**Keywords:** Evolution, Evolutionary ecology

## Abstract

Characterising how the totality of primate diversity is distributed across the order, and how it evolved, is challenging because diversity in individual traits often show opposing phylogenetic patterns. A species’ combination of traits can be conceptualised as its ‘niche’. Here, we describe and analyse seven-dimensional niche space, comprising 11 traits, for 191 primate species. Multifaceted diversity is distributed unequally among taxonomic groups. Cercopithecoidea and Hominidae occupy the largest areas of niche space, and are the most diverse families; platyrrhine families occupy small areas, and this space overlaps with strepsirrhines. The evolution of species’ locations in niche space is regulated by selection for adaptive optima in trait combinations. Given that niche similarity results in interspecific competition, we quantify two measures of species’ niche locations relative to others. We find that omnivores, frugivores, and species tolerating higher temperatures experience stronger interspecific competition. Hominidae occupation of niche space suggests competitive exclusion from niches by Cercopithecoidea over evolutionary time; but living great apes experience the lowest levels of interspecific competition. Callitrichids experience the highest levels of interspecific competition. Our results provide a standardised measure of primate niches that sheds light on the partitioning and evolution of primate diversity, and how this is driven by interspecific competition.

## Abstract (web summary)

7-dimensional niche space of 191 primate species shows niche diversity is partitioned unequally. Interspecific competition is lowest in great apes and highest in callitrichids.

## Introduction

Modern-day primates are a diverse group of mammals, with body masses ranging from 30 to 160,000 g (*Microcebus* and *Gorilla)*, diets spanning almost the entire range of mammalian diets (from e.g., grass-eating *Theropithecus*, to regular aquatic faunivory in *Macaca)*, and social systems ranging from solitary (e.g., *Pongo*) to multilevel groups (*Theropithecus)* and fission-fusion communities (*Pan*). Describing how this multifaceted diversity is distributed among the various phylogenetic groupings—for instance, are platyrrhines ‘less diverse’ than catarrhines?—has implications for conservation^1^ and evolutionary biology^2^, but is difficult to do because individual facets can show opposing patterns. For example, platyrrhines are characterised by low size diversity relative to other haplorhines but have diverse life history strategies and cooperative breeding systems^3^. This multifaceted diversity, furthermore, requires an evolutionary explanation. Models for the evolution of individual elements of this diversity have been successful with, for example, some between-species variation in female bonding, mating systems, or social structure being ultimately explained by a limited number of ecological variables^4^, or variation in long bone anatomy being explained by locomotor behaviour^3^. However, given developmental and ecological relationships between these elements^5–7^, they cannot evolve wholly independently. The first set of questions we are interested in here is how these elements relate to one another, how they are distributed across the Primate order, and how they evolve in relation to each other.

**Fig. 1:**
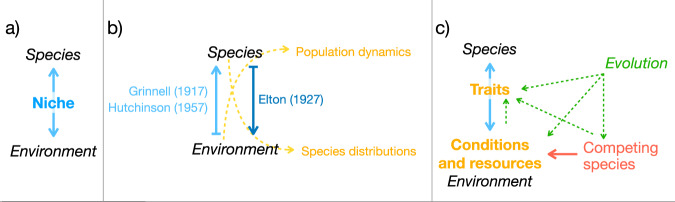
The concept of ‘niche’. **a** The underlying logic of most definitions of ‘niche’ is that it is an abstract sum of all relationships between a species and its environment. Blue colours represent the niche of a single species throughout the figure; **b** Formal definitions of the niche differ in their emphasis on processes and directions of processes that comprise these relationships. The yellow text represents measurable variables throughout the figure; **c** species’ intrinsic traits mediate how a species relates to conditions and resources in its environment and are therefore an important determinant of niches. Competing species regulate the availability of resources; and the actual niche a focal species occupies is consequently determined by interspecific competition (Hutchinson, 1957). The three factors that play a major role in determining a species’ niche are subject to evolutionary pressures (indicated by dashed green arrows). Species’ traits are subject to both abiotic evolutionary pressure (indicated by the dashed green arrow from the environment to traits) and biotic evolutionary pressure (indicated by the dashed green arrow from competing species to traits). Species’ occupied niches, then, are the outcome of the aggregate of these evolutionary processes.

A species’ ‘niche’ incorporates the totality of its behavioural, morphological, and ecological attributes. Quantifying these in a standardised way, therefore, offers a single comparative measure comprising all elements of diversity that can be used to describe the relationships among these attributes but also characterise the distribution of diversity across the clade and to explore the evolution thereof. At the most abstract level, all definitions of the niche describe a species’ specific relationship to an ecosystem^8^ (Fig. 1a). More specifically, the niche is usually used to describe the totality of ecological processes a species is involved in, such as responses to abiotic conditions, feeding, predator-prey interactions, mutualisms, and interspecific competition. The principal difference between formal definitions of niches is an emphasis on different types or directions of processes governing a species’ relationship to ecosystems (Fig. 1b). For example, Grinnell^9^ and Hutchinson^10^ stressed a species’ ecological requirements—that is, the scenopoetic conditions and bionomic resources a species *needs* from the environment. Elton^11^, on the other hand, emphasised a species’ ecological role within ecosystems—what a species *does* within it. What remains true across all niche definitions is that a focal species’ relationships to its environment—its niche—comprises two facets (Fig. 1c): (1) the conditions and resources available and (2) its own intrinsic traits, which define its ecological requirements and its potential responses to environmental conditions and resources. If these are quantified in a standardised way across species, their location within the ‘niche space’ generated captures these two facets.

Despite the pervasiveness and analytical appeal of the niche concept, how to translate formal definitions of niches into measurable practice has been the subject of intense theoretical debate^12^. A common solution to the problems presented by the complexity and sheer number of processes involved in ‘the niche’ is inferring niches from patterns that the implicated processes generate (Fig. 1b). Examples of such proxy-based approaches, first, are species distribution models (SDMs)^13,14^. These infer niches based on species occurrence data at the scale of the environment, arguing that the interactions between the three principal determinants of niches must underlie species’ geographical distributions. Second, the conditions required by a species—a Grinnellian or Hutchinsonian niche concept—are often approximated by linking species’ population growth dynamics to variation in an environmental variable of interest^15^. Rather than a proxy-based approach, the two niche factors—intrinsic traits and ecological conditions—can be explicitly modelled, and this is the approach we take here. When this has been done in previous work, the complexity of ecosystem dynamics has frequently necessitated restrictions to subsets of taxonomic diversity and/or variables^16^. Indeed, work on the quantification and evolution of individual primate niches has principally focused on the evolution of individual factors. Phylogenetic signal in climatic niche features is low^17^, suggesting that primates are less likely to ‘inherit’ these niche features from parent species. In line with these results, climatic niche evolution in primates does not occur at a constant rate and is instead characterised by clade-specific shifts in evolutionary rates^18^. These patterns do not seem to hold for intrinsic traits, however: primate body mass evolution conforms to a Brownian motion model^19^ and primate brain and body mass show high phylogenetic signal^20^, suggesting these evolve under a constant-rate regime. However, what evolutionary mode characterises the concurrent evolution of multiple facets of primate diversity—that is, primate niches—is poorly understood.

A species’ location within niche space is also determined by a third variable: other species, which interact with the focal species and the environment^8,12^. A particularly important evolutionary process generated by (the niches of) other species is interspecific competition. Indeed, Hutchinson^10^ formally differentiated between fundamental niches—the potential space a species could occupy in an *n-*dimensional space described by all environmental variables relevant to its survival—and realised niches—the actual niche space a species is restricted to by competition for similar resources with other species. Consequently, the topology of niche space should reflect interspecific competition. A niche measure based on four variables (diet, locomotion, activity cycle, and body mass) for sympatric species at eight localities showed that niches of these primates are best described in a ten-dimensional space, that South American species are more tightly clumped than those within any of the other communities, and that primate communities *within* regions exhibited greater ecological niche similarity than *between* regions^21^. This suggests high levels of interspecific competition within communities, particularly within South American communities, and raises the question of how this is mediated at local scales. Fleagle and Reed^21^ noted that no species filled the inner areas of community niche space and raised the possibility that this reflects within-community partitioning of niche space. The mechanisms by which niche space might be partitioned include fine-grained dietary divergence and the use of different locomotor repertoires. Chemical and stable isotope analyses suggested that primates generally conform to the competitive exclusion principle: sympatric primates tend *not* to occupy similar dietary niches^22^. Schreier et al.^23^ also suggested, based on a global sample of sympatric species pairs, that sympatric primates avoid direct interspecific competition by separating dietary niches and partitioning physical space. Whether the patterns Fleagle and Reed^21^ found within this small subset of primates hold across the Order more generally is unknown. The second goal of this paper, then, is to use our standardised measure of primate niches to comment on phylogenetic patterns in past and present interspecific competition. Specifically, we aim to identify species, or groups of species, that are likely subject to high levels of interspecific competition in the present and those whose present-day location in niche space may be explained by interspecific competition in the evolutionary past.

Here, we quantify the locations of 191 primate species in a multidimensional niche space. To do so, we use 11 variables—hereafter, ‘niche elements’—comprising *both* measurable facets of niches: intrinsic traits, conditions and resources. We then calculate phylogenetic signal and run models of evolutionary modes to ask how species’ locations across individual niche dimensions evolve. To shed light on primate interspecific competition, we quantify two measures of species’ locations *relative* to others in niche space: uniqueness (species’ distance to closest *neighbours* in niche space) and specialisation (species’ distance to the *centroid* of the niche space)^24^. Given that species with similar niches are expected to experience strong interspecific competition—an axiomatic principle in niche concepts^25^—high levels of clustering, small areas of niche space occupied, and few outliers in niche uniqueness and specialisation imply high levels of niche competition in the present. Species with low uniqueness scores, further, likely experience the highest levels of niche competition. By contrast, past interspecific competition should drive competing species’ niches away from each other, leaving the opposite topological signatures. Our results provide a quantified and standardised measure of primate niches that sheds light on the partitioning and evolution of primate diversity and the role of interspecific competition therein.

## Results

### The multidimensional niche space of extant primates

**Fig. 2:**
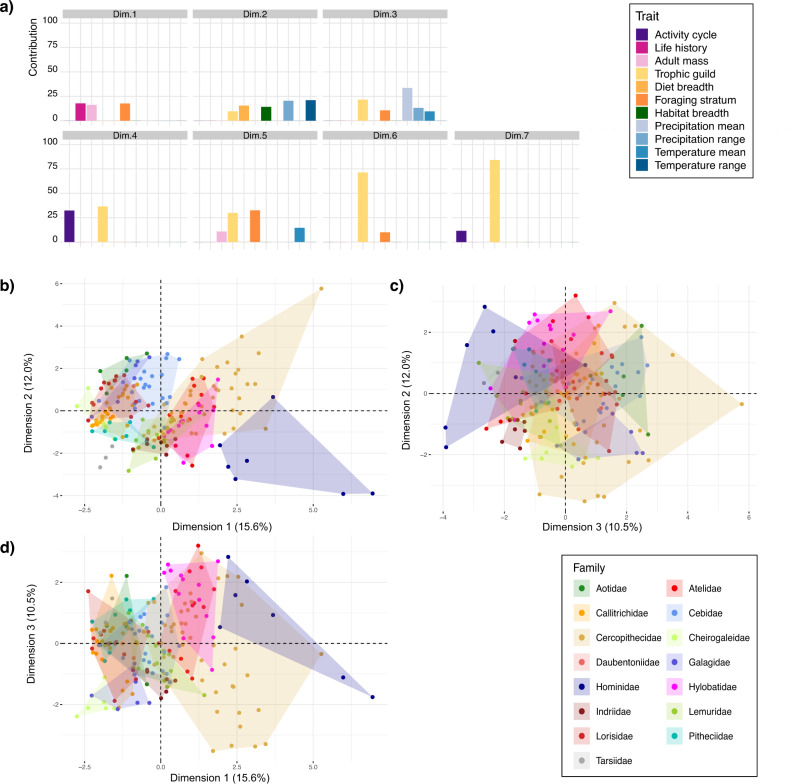
The multidimensional niche space of the Primate order. **a** The percentage contributions to each niche dimension of traits with >9% contributions. **b**–**d** Distribution of primate species, grouped by family, in (**b**) dimensions 1 and 2, **c** dimensions 2 and 3 and **d** dimensions 1 and 3 of the 7-dimensional niche space. 3D model of the first three dimensions available on the Figshare repository.

The FAMD indicated that variation in the complete set of niche elements is best explained in a reduced, seven-dimensional niche space that explains 67.7% of the variation (Supplementary Figs. 1 and 2). This space is referred to as ‘niche space’ hereafter. Niche elements contributing to each dimension are presented in Fig. 2a.

Species’ locations along the first three dimensions are visualised in Fig. 2b–d. These are available as a 3D plot on the Figshare repository. The occupation of higher-level taxonomic groupings are presented in Supplementary Figs. 3 and 4.

The first dimension of the niche space explains 15.8% of the total variation and is predominantly associated with species’ intrinsic traits: life history, adult body mass, and foraging stratum (Fig. 2a). Most primates are positioned closely together, and families show some overlap along this first dimension, with a notable set of outliers: Hominidae is comparatively separated from the other primates, and only overlap along this axis with some members of Cercopithecidae (Fig. 2b). The second dimension explains 12.0% of the total variation and is a mixture of Eltonian and Grinnellian/Hutchinsonian niche concepts, comprising dietary traits (trophic guild, dietary breadth) and environmental variables (habitat breadth, precipitation range, temperature range). Along this axis, again, Hominidae is separated from other primates, but not to the same degree as was the case along dimension 1. Across dimensions 1 and 2 together, however, Hominidae occupies a comparatively isolated range of primate niche space. The third dimension comprises trophic guild, foraging stratum, precipitation mean, temperature mean, and temperature range and explains 10.5% of the total variation. Together characterising 29.4% of the total niche variation, the other four dimensions all comprise the foraging stratum and species’ intrinsic traits (Fig. 2a).

A striking result is the degree of overlap between the niches of primate families (Fig. 2b–d): most families overlap almost completely with others, and most primate niches, then, are comparatively similar to those of others. Despite this general pattern of overlap, there are some trends in the occupation of niche space. Cercopithecoidea occupies the largest area of the overall niche space, and its location is comparatively central across all dimensions. Hominidae also occupies a large area of niche space, but its location is more separated from that of other primates. Finally, in dimensions 1 and 2, there are two groups composed of phylogenetically disparate families: one comprising Cercopithecidae, Atelidae, Lemuridae, Indriidae, Galagidae, and Hylobatidae, and the other comprising Aotidae, Callitrichidae, Lorisidae, Tarsiidae, Cheirogaleidae, and Pitheciidae. Cebidae and Hominidae both occupy similar positions relative to groups one and two, respectively, with some overlap but higher scores for dimension one. Finally, there is near complete overlap in niche space occupied by Hylobatidae and Atelidae.

### Niche uniqueness and specialisation: species’ relative locations in niche space

Niche uniqueness and specialisation scores for all species included in the analyses are presented in Supplementary Data 1. Niche uniqueness and specialisation are rare in the primate order: there are 8 outliers for uniqueness and 9 outliers for specialisation (Supplementary Fig. 5). The top ten most unique and specialised niches span 6 families (Tables 1 and 2). The pygmy slow loris (*Nycticebus pygmaeus*), common marmoset (*Callithrix jacchus*), eastern gorilla (*Gorilla beringei*), rhesus macaque (*Macaca mulatta*), western gorilla (*Gorilla gorilla*), and gelada (*Theropithecus gelada*)) are amongst the top 10 most ecologically unique and specialised. The eastern gorilla and pygmy slow loris both appear in the top three for both metrics, suggesting their niches are highly distinct compared to the rest of the order. The niche of the rhesus macaque is the fourth most unique and specialised, a pattern likely explained by its exceptionally large geographic range and dietary flexibility^3^.

**Table 1:** Top 10 highest niche specialisation scores

**Top 10**	
1	*Gorilla beringei*
2	*Nycticebus pygmaeus*
3	*Gorilla gorilla*
4	*Macaca mulatta*
5	*Callithrix jacchus*
6	*Cephalopachus bancanus*
7	*Tarsius tarsier*
8	*Tarsius fuscus*
9	*Theropithecus gelada*
10	*Papio anubis*

**Table 2:** Top 10 highest and lowest niche uniqueness scores

Top 10 (lowest competition intensity)		Lowest 10 (highest competition intensity)		Lowest 10 (platyrrhine-only analysis)	
1	*Nycticebus pygmaeus*	191	*Leontocebus fuscicollis*	65	*Mico humeralifer*
2	*Callithrix jacchus*	190	*Cebuella niveiventris*	66	*Mico argentatus*
3	*Gorilla beringei*	189	*Saguinus mystax*	67	*Saguinus midas*
4	*Macaca mulatta*	188	*Leontocebus cruzlimai*	68	*Mico leucippe*
5	*Gorilla gorilla*	187	*Leontocebus illigeri*	69	*Leontocebus fuscicollis*
6	*Theropithecus gelada*	186	*Mico leucippe*	70	*Leontocebus illigeri*
7	*Daubentonia madagascariensis*	185	*Leontocebus nigrifrons*	71	*Leontocebus nigrifrons*
8	*Pan troglodytes*	184	*Saguinus midas*	72	*Cebuella niveiventris*
9	*Loris tardigradus*	183	*Mico humeralifer*	73	*Leontocebus cruzlimai*
10	*Semnopithecus entellus*	182	Leontocebus tripartitus*	74	*Saguinus mystax*

*Not present in the lowest-scoring 10 in the platyrrhine-only analysis.

Cercopithecidae and Hominidae are overrepresented in the top 10 most unique and specialised rankings, with three cercopiths and three great apes in the top ten most unique species, and three cercopiths and two apes in the top ten most specialised species. Hominidae contributes substantially more species to these lists (42% of Hominidae are represented in the top 10 unique species and 29% in the top 10 specialised species). Additionally, there are two lorises that score highly for uniqueness, and three tarsiers in the top ten most specialised species.

Species likely undergoing the lowest competition intensity then comprise 42% of Hominidae (Table 2). Species with the lowest niche uniqueness scores follow a very different phylogenetic pattern: the top 10 lowest-scoring species all belong to Callitrichidae. Since platyrrhines are the only South American primate group, and there is overlap between the niche space occupied by platyrrhines and other primates, it may be the case that the high competition intensity we infer for callitrichids on the basis of the whole sample is misleading, so we repeated our analyses for a subset of the data restricted to platyrrhines. All but one species (*Leontocebus tripartitus*) in the lowest-scoring 10 of this subsample are also included in the lowest-scoring 10 of the overall primate sample, so the high levels of competition inferred for callitrichids is not an artefact of overlap in niche space with non-sympatric species.

Beyond these lowest 10, the lowest 20% of the overall primate sample include members of (in order of first appearance) 5 families: Pitheciidae (3), Lorisidae (4), Hylobatidae (5), Cebidae (6), and Cercopithecidae (4). Callitrichidae are overrepresented in the top 20% lowest-scoring species, with a total of 16 species (50% of the total lowest-scoring 20%) included.

The largest positive effect on niche uniqueness score in phylogenetic generalised least squares (PGLS) models run across 100 primate phylogenies was from gummivory: compared to a reference level of folivory, it produced a median increase in niche uniqueness score across all phylogenies of 0.7299 (Table 2a). Gummivory, then, appears to be a major factor reducing interspecific competition in primates. Other niche elements that significantly increased a species’ niche uniqueness score across more than 50% of phylogenies were scansorial foraging (compared to arboreal foraging), larger habitat breadths, and increased precipitation range. Niche elements that were significantly associated with a reduction in niche uniqueness score across all phylogenies, and thus likely act to increase interspecific competition, were higher mean temperature and omnivory (compared to a reference level of folivory). Other niche elements that significantly decreased a species’ niche uniqueness score across more than 50% of phylogenies were folivory-frugivory and frugivory (both compared to a reference level of folivory).

Niche elements that were positively associated with niche specialisation score across all phylogenies were gummivory (compared to a reference level of folivory), which had the largest effect at 0.4789, and larger habitat breadth. The other niche element that significantly increased a species’ niche specialisation score across more than 50% of phylogenies was ground foraging (compared to arboreal foraging). All compared to a reference level of folivory, frugivory, omnivory, and folivory-frugivory had strong negative effects on niche specialisation score across all phylogenies; and higher mean temperatures did so too, across 91% of phylogenies.

### Exploring evolutionary dynamics: how do species’ individual locations across individual niche dimensions evolve?

**Fig. 3:**
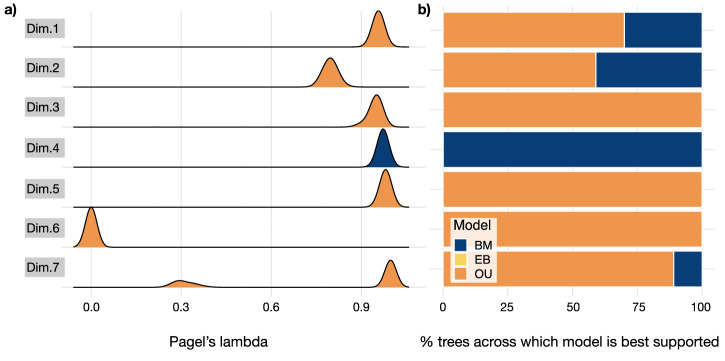
The evolution of species’ positions across niche dimensions. **a** Distribution of Pagel’s lambda for each niche dimension, coloured by the best-supported model of evolutionary mode (BM Brownian motion, blue; EB early burst, yellow; OU Ornstein-Uhlenbeck, orange). **b** The percentage of trees across which model of evolutionary mode is best supported for each niche dimension. Dim. dimension.

Phylogenetic signal estimates using Pagel’s λ indicated strong and statistically significant phylogenetic signal in nearly all the niche dimensions (Fig. 3a). Across the 100 trees, dimensions one, three, four, five, and seven showed strong phylogenetic signal (median *λ* = 0.95, 0.95, 0.97, 0.98, and 0.99, respectively), meaning variation between species’ locations across these dimensions can be almost completely explained by shared evolutionary history—in other words, more closely related species are more closely positioned across these dimensions. Dimension six, which is a close approximation of Eltonian facets of a niche, as it comprises trophic guild and foraging stratum, is characterised by extremely low phylogenetic signal (median *λ* = 0). Closely related species, then, do not tend to be characterised by similar trophic guilds or foraging strata.

In-keeping with previous work showing that the link between phylogenetic signal and evolutionary processes is complex^26^, dimensions across which high phylogenetic signal was inferred did not tend to be best described by Brownian motion models in the maximum likelihood model fitting (Fig. 3b); whilst for dimension 6, which is characterised by low phylogenetic signal, Brownian motion models were selected across 100% of the phylogenies. The Brownian motion model was selected across all phylogenies for dimension 4, which uniquely comprises activity cycle. For all other dimensions, Ornstein-Uhlenbeck models were selected across >50% of the trees, suggesting species’ locations along these dimensions evolved in a regime characterised by adaptive optima. Early burst models were never selected across any tree for any dimension.

## Discussion

The niche space of the Primate order is composed of seven dimensions, each of which comprises several types of niche elements. In total, these seven dimensions explain 67.7% of the variation in primate niche elements. Overall, there is little differentiation between primate families’ niches: there is extensive overlap between families in all dimensions (Fig. 2b–d). Most primate species, in other words, occupy a similar niche. This average primate niche is defined by the niche elements that contribute negatively to the uniqueness and specialisation scores: tolerating relatively high mean temperatures, *not* being gummivorous or folivorous, and tolerating a small range of habitats. The exceptions to this general pattern are the catarrhines, who are comparatively disparate from other groups across dimensions one and two and one and three.

The high levels of niche similarity across the Primate order we find here may be explained by the comparatively large geographical range occupied by the order. It may be the case, for example, that there are actually high levels of niche separation at smaller scales between sympatric species and that this signal is lost when samples are pooled. However, there are no obvious geographic patterns in families’ occupation of niche space to support this hypothesis—that is, families that occupy the same biogeographic realm do not occupy different sections of niche space. The combined pattern of low numbers of niche specialisation and uniqueness outliers and pattern of sympatric families overlap in niche space implies high levels of ecological redundancy (*sensu*^24,27^). This may buffer the functionality of primate communities in the face of extinction. Because primates tend to occupy very similar niches, the extinction of one will not immediately remove an entire set of functions it provides.

Despite the overall lack of nonoverlapping areas of niche space occupied by primate families, primate species do not occupy the exact same position in niche space. Primates partition niche space primarily through differentiation in adult body size, life history, and foraging stratum—the principal contributing elements of niche dimension one, which explained 15.6% of the overall variation (Fig. 2a). That adult body size and life history are principal contributors to the first niche dimension is unsurprising, given that these correlate with most aspects of physiology^5,28^, and are thus a critical determinant of how species interact with environments. The presence of a foraging stratum as a contributor to this dimension is in line with previous work, which suggested that the most important means by which African and Asian sympatric primates partition niche space is the use of different foraging strata^23^.

Trophic guild is a contributor to all six further niche dimensions (Fig. 2a), again in keeping with previous work showing that dietary differences are an important way in which niche space is partitioned^23^. Of these six niche dimensions, dimensions two and three together explain an additional 22.5% variation in niche elements. The combination of dietary (Eltonian) and climatic (Grinellian/Hutchinsonian) variables across these two dimensions—rather than a clear separation of dietary and climatic variables between the two dimensions—highlights the interrelatedness of these niche elements (Fig. 1c) and is reminiscent of longstanding conceptual confusion about how these influential niche definitions differ^29^. In other words, our results suggest specific Eltonian and Grinellian/Hutchinsonian niche elements evolve as combinations rather than independently and that this interrelatedness goes some way to explaining the persistence of debate about how to best translate the concept of niche to quantified practice^12^.

Although we incorporate multidimensionality in our approach, ours differs from that of Hutchinson’s^10^ in that our data are composed of average estimates for each trait rather than the range of values under which a species can survive; and our niche axes comprise combinations of traits, thus taking into account interactions between them^29^. For the climatic elements, our method assumes mean temperature and precipitation accurately reflect that of a species’ niche source—representing conditions the species thrive in, as opposed to sinks in which populations could not exist without immigration from sources^30^. This may not be the case, and these estimates may be particularly influenced by anthropogenic effects, but the effect on our data is most likely randomly distributed given the global scale of anthropogenic habitat disturbance and climate change.

The niches modelled here approximate realised niches rather than fundamental niches^10^. Many primate species are experiencing geographic range restriction due to anthropogenic activity^31^, and this may have affected foraging and climatic variables. We cannot explicitly correct for the effect of anthropogenic impacts. However, that other species—including *Homo sapiens—*play a major role in determining a species’ relationships to its environment is implicit in our and most formal definitions^11,12,16^, of a species’ realised niche (Fig. 1c). Furthermore, the consequences of the global scale of anthropogenic habitat disturbance^32,33^ is that its effect on our data is going to be practically randomly distributed—and therefore exert little influence on the taxonomic patterns in the occupation of niche space.

That our approach accurately quantifies niches described but not formally quantified in previous work is evidenced in, for example, the near-complete overlap in niche space occupied by the phylogenetically and geographically disparate brachiator families Atelidae and Hylobatidae (Fig. 2b–d). Brachiation is typically described as uniquely having opened the below-branch feeding niche to these two groups^3,34–37^. Despite not including the trait—locomotion—that allowed access into this space within the niche landscape, our method identified it.

Multifaceted primate diversity is not distributed equally among taxonomic groups. Cercopithecidea and nonhuman Hominidae (hereafter ‘Hominidae’) occupy the largest areas of primate niche space (Fig. 2b–d), meaning that these two families are the most comparatively diverse groups of primates. This is particularly interesting in the case of Hominidae because its comparatively large niche diversity contrasts with its taxonomic paucity (7 species). Each great ape species, then, represents a proportionally greater range of niche diversity, underscoring calls for directed conservation efforts towards these taxa^38^.

Despite its comparatively large size (Fig. S5), the space occupied by catarrhines does not overlap extensively with that occupied by all other primates across dimensions one and two and one and three. Modern-day catarrhines, our results suggest, radiated into a distinct area of niche space. Applying our method to fossil samples will shed light on whether this space was once occupied by fossil non-catarrhines or whether key catarrhine innovations uniquely permitted access to unoccupied niche space.

In contrast, Platyrrhine families each occupy comparatively small areas, and Plattyrrhini overall occupies a smaller area than Catarrhini. This space, further, overlaps with those of Lemuroidea and Lorisoidea (Fig. S5). The comparatively small area occupied by Platyrrhini is surprising, given that they are the only primate radiation found within South America and thus presumably should have radiated into diverse and empty niche space^3^. The comparative lack of niche diversity captured by the high degree of clumping of Platyrrhini within niche space aligns with the high levels of clumping in this group found in Fleagle and Reed’s^21^ analysis, which described two dimensions of primate niche space. Terborgh and van Schaik put forward ecological constraints on body size, particularly high levels of seasonality and overlap between fruiting and leafing times, as an explanation for the low levels of niche diversity in platyrrhines^39^; others have suggested interspecific competition with sloths limited platyrrhine diversity^21^.

The evolution of species’ locations along all dimensions but dimension four is best explained by Ornstein-Uhlenbeck models, which incorporate adaptive optima (Fig. 3b). All six of these dimensions comprise varying combinations of niche elements. Taken together, these results imply that primate evolution was regulated by the pull towards specifically adaptive *combinations* of niche elements rather than the selection on these individually. This result is in line with previous work showing that primate climatic niche evolution does not occur at a constant rate and is instead characterised by clade-specific shifts in evolutionary rates^18^. Our results show that this dynamic also characterises the evolution of other niche elements and, crucially, that these evolve towards adaptive optima in trait combinations.

Intriguingly, all but one of the dimensions across which Ornstein-Uhlenbeck models were inferred had consistently high phylogenetic signals, suggesting closely related species tend to have a similar trait combination. This implies that, generally, selection towards adaptive optima in trait combinations contributed towards primate diversification *above* the species level. The exception to this pattern is dimension six, to which the trophic guild is a particularly important contributor alongside the foraging stratum. Closely related species, then, do not tend to have similar combinations of trophic guild and foraging stratum. An implication of this result is that these traits should be disproportionately involved in speciation. Dimensions two and three, which comprise a greater number of climatic niche elements than other dimensions, are characterised by comparatively high phylogenetic signals. This contrasts with results from previous studies, which found weak phylogenetic signals in climatic niche elements^17,20^. This difference may be explained with reference to methodology: these studies calculated phylogenetic signal using Blomberg’s K—rather than Pagel’s lambda—and for individual climate variables—rather than combinations thereof.

Dimension four, which comprises the activity cycle and trophic guild, is the only dimension for which there is strong support that the evolution of species’ locations along it was *not* driven by selection towards adaptive optima. Instead, Brownian motion best explains the evolution of combinations of different activity cycles and trophic guilds (Fig. 3a). This result suggests an absence of strong selection on activity cycle and trophic guild combinations (but see^40^).

Niche elements contributing negatively to niche uniqueness, and thus characterising species that likely experience higher levels of interspecific competition, were tolerating higher mean temperatures, being omnivorous, folivorous-frugivorous, and frugivorous (compared to folivorous), This builds upon previous work which, although based on a very different analytical approach, identified frugivores as experiencing higher levels of interspecific competition than folivores^41^. Niche elements that are principally involved in isolating species within niche space—and thus reducing the pressure of interspecific competition—are gummivory (compared to folivory), scansoriality (relative to arboreality), and tolerating a broad range of habitats. Thus, we found that the traditional ecological definition of generalism—that is, thriving in a wide range of habitats—is a strategy that contributes to minimising interspecific competition.

In dimensions 1 and 2, there are two groups composed of phylogenetically disparate families: one comprising Cercopithecidae, Atelidae, Lemuridae, Indriidae, Galagidae, and Hylobatidae, and the other comprising Aotidae, Callitrichidae, Lorisidae, Tarsiidae, Cheirogaleidae, and Pitheciidae. Hominidae and Cebidae both occupy similar positions relative to groups one and two, respectively, with some overlap but higher scores for dimension one. This is interesting, given that primate tool use is phylogenetically concentrated in these two families^42–44^, and likely plays an important role in these groups’ ecological strategies. It may be the case that the niche space opened up by tool use reduces interspecific competition. The lowest levels of interspecific competition are, indeed, inferred for Hominidae. Great ape species occupy a large area of niche space despite the comparative taxonomic paucity of this group. Great ape species, further, are overrepresented in the top 10 highest-scoring species for niche uniqueness.

Great ape niches are located comparatively far from those of other primates, especially across dimensions one and two together. The closest to great ape niche space is that occupied by Cercopithecoidea—and the topology of these families’ niche locations hints at competitive dynamics, as it is congruent with competitive exclusion. The isolation of the extant great ape clade in the context of known fossil ape diversity suggests that extant great apes were competitively excluded by cercopiths from more generalist and non-unique niches once occupied by Miocene hominids. This is the pattern predicted by Hunt^45^, who hypothesised that hominids evolved suspensory behaviour in response to being outcompeted for fruit by the expanding Cercopithecoidea lineage. If non-random Hominidea extinctions resulting from competition with cercopith species underlie this pattern, this explains the overrepresentation of great apes among species scoring highly for niche uniqueness and specialisation.

Our results suggest Platyrrhines, and particularly callithrichids, experience the comparatively highest levels of interspecific competition in the present: the top 10 lowest niche uniqueness scores were found in this subgroup. To the best of our knowledge, this has not been documented in previous work. This is a surprising result in the face of the role of gummivory in reducing the strength of interspecific competition across the order overall (Table 3a). However, this effect is likely driven by the high uniqueness scores of the plant exudate-eating pygmy slow loris (*Nycticebus pygmaeus*), common marmoset (*Callithrix jacchus*), and aye-aye (*Daubentonia madagascariensis)*. The high levels of interspecific competition inferred for callitrichids are also surprising in the context of their habitual mixed-species associations^46^ and raise the question of how negative fitness associated with interspecific competition is avoided in these groups. The partitioning of vertical space and attendant fine-grained differences in diet have been documented for these mixed-species groups^46^. Fine-grained dietary differentiation has also been argued to underlie hand morphology specialisations in callitrichids^47^. Rylands, furthermore, linked the unique breeding systems in callitrichids to fine-grained differences in habitat use and diet^48^, which, our results suggest, are underlain by high levels of interspecific competition. Our results suggest these unique callitrichid adaptations and fine-grained dietary specialisations may be the consequences of high levels of interspecific competition. However, the intriguing case of dietary niche expansion, rather than specialisation, by Goeldi’s monkey (*Callimico goeldii*) in mixed-species groups underlines the diversity of potential adaptive strategies in response to interspecific competition and exploring the dynamics and consequences of interspecific competition in callitrichids is a clear direction for future work raised by our results.

**Table 3:** Model outputs from PGLS analysing which niche variables contribute to uniqueness and specialisation scores, controlling for phylogeny

Effect	Niche variable	Median estimate	Significant adjusted p-values count across 100 phylogenies	Median *p*-value
*Uniqueness score*				
+	Gummivory	0.7299	100	0.0000*
	Intercept	0.2914	0	0.0537
+	Ground foraging	0.1540	31	0.0072*
+	Scansorial foraging	0.1252	81	0.0015*
	Insectivory	0.1130	0	0.2770
	Cathemerality	0.0852	0	0.3882
+	Adult mass	0.0483	34	0.0069*
+	Habitat breadth	0.0313	99	0.0000*
+	Precipitation range	0.0247	96	0.0001*
	Mean precipitation	0.0049	1	0.3414
	Life history	0.0036	0	0.6798
	Temperature range	0.0031	1	0.4634
	Diet breadth	0.0006	4	0.5550
−	Mean temperature	−0.0327	100	0.0000*
−	Folivory-frugivory	−0.0554	79	0.0004*
	Diurnality	−0.0807	0	0.3653
−	Frugivory	−0.0883	99	0.0000*
−	Omnivory	−0.1497	100	0.0000*
*Specialisation score*				
+	Intercept	0.5558	34	0.0060*
+	Gummivory	0.4789	100	0.0000*
+	Ground foraging	0.2354	73	0.0020*
	Cathemerality	0.1101	0	0.4080
	Scansorial foraging	0.0706	0	0.1810
+	Adult mass	0.0685	48	0.0050*
+	Habitat breadth	0.0439	100	0.0000*
	Insectivory	0.0381	0	0.7810
+	Precipitation range	0.0207	25	0.0120*
+	Diet breadth	0.0180	3	0.0400*
	Life history	0.0178	0	0.5840
	Mean precipitation	0.0142	14	0.0840
	Temperature range	−0.0063	2	0.3840
−	Mean temperature	−0.0269	91	0.0000*
	Diurnality	−0.0744	0	0.5390
−	Folivory-frugivory	−0.2269	100	0.0000*
−	Omnivory	−0.2974	100	0.0000*
−	Frugivory	−0.3182	100	0.0000*

+ indicates a significant and positive contribution to uniqueness or specialisation score. − indicates a significant and negative contribution to uniqueness or specialisation score.

* indicates that the median adjusted *p*-value was less than 0.05.

Medians were taken over all 100 primate phylogenies and rounded to 4 decimal places.

Elucidating the dynamics of extant competition for the general primate niche, but also the competition for adaptive optima in niche element combinations and competition between great apes and cercopiths over evolutionary time, can be done by applying our approach to fossil data of hypothetical niche competitors. The extinction of species with similar niches—which would be competitors if sympatric with the focal taxon—would increase the focal taxon’s uniqueness score by increasing its distance to its nearest neighbours and may also increase the focal’s specialisation score if its extinction has a strong effect on the location of the niche space’s centroid. The effect of interspecific competition is likely to be similar: axiomatic in niche concepts is the principle that sympatric species cannot occupy the exact same niche^25^, and interspecific competition, then, should drive competing species’ niches away from each other.

In summary, we quantified the niche landscape of the Primate order with a multidimensional measure comprising life history, morphology, behaviour, diet, and climate niche elements for 191 primate species. This landscape is composed of seven dimensions, each comprising unique combinations of niche elements. Multifaceted diversity is not distributed equally among taxonomic groups: Cercopithecoidea and Hominidae occupy the largest areas of primate niche space, meaning species belonging to these families represent more of overall primate diversity than species belonging to other families. In contrast, platyrrhine families each occupy comparatively small areas, and this space overlaps with that of strepsirrhines. Species’ locations across all but one niche dimension and in multidimensional space evolve under an Ornstein-Uhlenbeck process. This, in combination with generally high phylogenetic signal in all measures, suggests selection towards adaptive optima in trait combinations contributed towards primate diversification above the species level. Primate niches evolve, in other words, in regimes determined by adaptive optima for niche element combinations rather than for niche elements individually. We found that both niche specialisation and uniqueness are very rare in primates. Most primates occupy similarly generalised and ecologically redundant (i.e. non-unique) niches, which are characterised by relatively high mean temperatures, low habitat breadth tolerance, and *not* being gummivorous or folivorous. Hominidae space occupation conforms to a pattern expected under competitive exclusion from more generalist niches by Cercopithecoidea. However, extant great apes experience disproportionately low levels of interspecific competition. Finally, we found that extant callitrichids experience the highest levels of interspecific competition. Applying this method to extinct primates has the potential to clarify open questions about the role of niche competition in the evolution of the Primate order.

## Methods

### Data collection

We obtained data relating to primate niche elements, including life history, morphology, behaviour, ecology and climate. We extracted the following variables from the Amniote life history database^49^, accessed through the COMBINE database^50^: six life history traits measured in days (longevity, female maturity, gestation length, interbirth interval, weaning age and generation length) and adult body mass (in grams). Foraging stratum (ground level, scansorial or arboreal); activity cycle (nocturnal, diurnal, or cathemeral); and diet breadth (number of dietary categories, comprising more than 20% of a species’ diet) were extracted from the EltonTraits database^51^, accessed through the COMBINE database^50^. Activity cycle classifications were cross-checked and updated with the current IUCN Red List of Threatened species (https://www.iucnredlist.org; last accessed 2 November 2023)^52^. We collected each species’ trophic guild (folivore, frugivore, folivore and frugivore, omnivore, gummivore or insectivore) from the Ecological Traits of the World’s Primates database^53^, and any gaps were filled using IUCN Red List data and mammalDIET^54^. Supplementary Table 1 provides definitions of variables extracted from published databases. Habitat breadth, defined as the number of IUCN Red List level 2 habitat types deemed suitable for a given species, was collected from the IUCN Red List^52^. We obtained climatic niche data using spatial analysis methods in QGIS Version 3.14^55^. We used bioclimatic data from WorldClim version 2 at a spatial resolution of 2.5 minutes^56^. We took primate geographical ranges from the IUCN Red List spatial data^52^ and extracted the mean and range of both temperature and precipitation, as well as elevation range, within each species’ geographical range, using the ‘zonal statistics’ tool in QGIS. Species with any missing or imputed data were removed from analyses. The final dataset consisted of values for 16 niche traits for 191 species.

### Modelling primate niches

All analyses were carried out in the R environment^57^. To prepare the trait data for Factor Analysis of Mixed Data (FAMD, see below), we first examined the correlations between traits (Supplementary Fig. 6a). The six life history variables were highly correlated (Pearson’s *r* > 0.6), other than gestation length and maximum longevity (Pearson’s *r* = 0.49). Highly correlated variables are likely to dominate the contribution to the principal components^58^, so we carried out a Principal Component Analysis (PCA) on the six life history variables. Principal component 1 of this analysis explained 80.6% of the variance in life history across species. We added the coordinates of the first principal component to the niche trait dataset, to represent the life history component of a niche in one metric. In this principal component—referred to hereafter as ‘life history score’. We removed the six original life history variables from the dataset, leaving eleven niche elements.

Using the *FAMD* function of the ‘FactoMineR’ package^59^, we then carried out a factor analysis of mixed data on the niche elements. This method can use both categorical and continuous data to describe the variation in a dataset, potentially in a lower-dimension space. Additionally, this function automatically standardises the quantitative variables before analysis. When using categorical variables in this type of analysis, the same trait can contribute to several dimensions due to the multiple categories held within it^60^. We defined primate niche space as the space described by all dimensions with eigenvalues greater than one to comply with the Kaiser criterion (Supplementary Table 2). Primate niches, then, refer to the location of a species within this multidimensional space.

### Quantifying niche uniqueness and specialisation

We followed the definitions put forward by Mouillot et al.^24^, Leitão et al.^61^ and Pimiento et al.^27^, where uniqueness measures the isolation of a point relative to its nearest neighbours in multidimensional space, and ecological specialisation measures a point’s isolation relative to the centre of the space. We measured the mean Euclidean distance of each species’ niche location to its five nearest neighbours in multidimensional niche space to produce a niche uniqueness score for each species (i.e., the higher the score, the greater the mean distance from its nearest neighbours). Niche uniqueness, then, is a proxy for isolation of the focal species’ location in niche space and thus represents the inverse of redundancy (that is, the species does not have similar trait combinations to many others). To check that niche uniqueness scores were robust to the number of neighbours used in the calculation, we repeated the distance measurements with varying numbers of neighbours. The uniqueness score using the five nearest neighbours is strongly correlated with that using one (*p* = 0.85), three (*p* = 0.99), ten (*p* = 0.97) and 100 (*p* = 0.79) neighbours. Importantly, the top eight unique species—though their ranks differ slightly—are the same for five, ten, and 100 neighbours. Therefore, the measurement is robust to the number of neighbours used. The niche specialisation score was taken as the Euclidean distance of a species to the centroid of the multidimensionsional niche space^24,27,61,62^. Niche specialisation scores, then, capture how generalist (i.e., average combinations of niche elements, close to the centre of the space), or specialised (i.e., extreme combination of niche elements, far from the centre of the space) species are in niche space.

Niche uniqueness captures a species’ relative isolation in niche space. It can, therefore, be used as a measure of the intensity of interspecific competition, with species scoring highly likely not subject to strong competition from other primate species and low scores indicating a focal species’ niche is similar to those of other species. We report the top 10 highest and lowest-scoring species for niche uniqueness. This logic also means that niche factors positively contributing to niche uniqueness scores are those primates likely to use to avoid niche competition.

To assess which factors of a niche predict niche uniqueness and specialisation, we ran the following PGLS regression across the same 100 primate phylogenies:

$$S={{{{{\rm{intercept}}}}}}+{{{{{\mathrm{var}}}}}}_{1}+{{{{{\mathrm{var}}}}}}_{2}+{\ldots +{{{{\mathrm{var}}}}}}_{n}$$

where *S* is specialisation or uniqueness score, scaled to have a range between zero and one, and *var* is niche variable (i.e., body mass, diet breadth, trophic guild, foraging stratum, activity cycle, habitat breadth, precipitation mean, precipitation range, temperature mean, temperature range, and life history). Continuous variables were centred and scaled to have unit variance. For each primate phylogeny, the *p*-values associated with each niche variable were adjusted for multiple testing using the Holm–Bonferroni method. We then counted the number of significant adjusted *p*-values across all 100 phylogenies for each niche variable.

### Exploring evolutionary dynamics: how do species’ individual locations across individual niche dimensions evolve?

We first estimated the phylogenetic signal of species’ locations in each niche dimension across 100 randomly selected primate node-dated DNA-only phylogenies from VertLife^63^. Discrepancies between the taxonomies utilised by the IUCN Red List and VertLife were resolved using the IUCN Red List synonyms. We, therefore, estimated the phylogenetic signal of primate niches using Pagel’s *λ*. We used the *phylosig* function in the ‘phytools’ package^64^. Likelihood ratio tests were used to evaluate if niche distribution across the primates was significantly different than expected in a Brownian motion (BM) model (*λ* = 1) or a null model (no phylogenetic signal: *λ* = 0). Reported *p*-values are Holm–Bonferroni corrected.

We then fit three maximum likelihood models of the evolution of species’ locations across each niche dimension using the *fit_t_standard* function in the package ‘RPANDA’^65^ across the 100 randomly selected primate phylogenies:

1 $${{{{{\rm{Brownian\; motion}}}}}}:{{{{{\rm{\phi }}}}}}({{{{{\rm{t}}}}}})={{{{{{\rm{\sigma }}}}}}}_{0}^{2}$$

where *t* is time and ${{{{{\rm{\sigma }}}}}}$ is the ‘rate’ of the evolution of the species location (whether across a single dimension or its niche uniqueness/specialisation score);

2 $${{{{{\rm{Ornstein}}}}}}-{{{{{\rm{Uhlenbeck}}}}}}({{{{{\rm{OU}}}}}}):{{{{{\rm{d}}}}}}{X}_{t}=\alpha \left({{{{{\rm{\theta }}}}}}-{X}_{t}\right)+\sigma d{B}_{t}$$

where *t* is time, X_t_ is the species’ location (whether across a single dimension or its niche uniqueness/specialisation score) at time *t*, ${{{{{\rm{\sigma }}}}}}$ is the ‘rate’ of the evolution of a species location, $\alpha$ is the strength of selection, and ${{{{{\rm{\theta }}}}}}$ is the adaptive optimum species’ location;

3 $${{{{{\rm{Early\; burst}}}}}}:{{{{{\rm{\phi }}}}}}\left({{{{{\rm{t}}}}}}\right)={{{{{{\rm{\sigma }}}}}}}_{0}^{2}\times {e}^{-{rt}}$$

where *t* is time and ${{{{{\rm{\sigma }}}}}}$ is the ‘rate’ of the evolution of a species’ location (whether across a single dimension or its niche uniqueness/specialisation score), which declines through time as a function of rate *r*.

Model fits were compared using the small-sample corrected Akaike Information Criterion (AICc) weights, with the model with the lowest AICc score being selected as the best fit.

### Reporting summary

Further information on research design is available in the Nature Portfolio Reporting Summary linked to this article.

### Supplementary information

Peer Review File

Supplementary Information

Description of Additional Supplementary Materials

Supplementary Data 1

Reporting summary

## Data Availability

All data is publicly available on the Figshare database (10.6084/m9.figshare.25721466).

## Code availability

All code is publicly available on the Figshare database (10.6084/m9.figshare.25721466).
