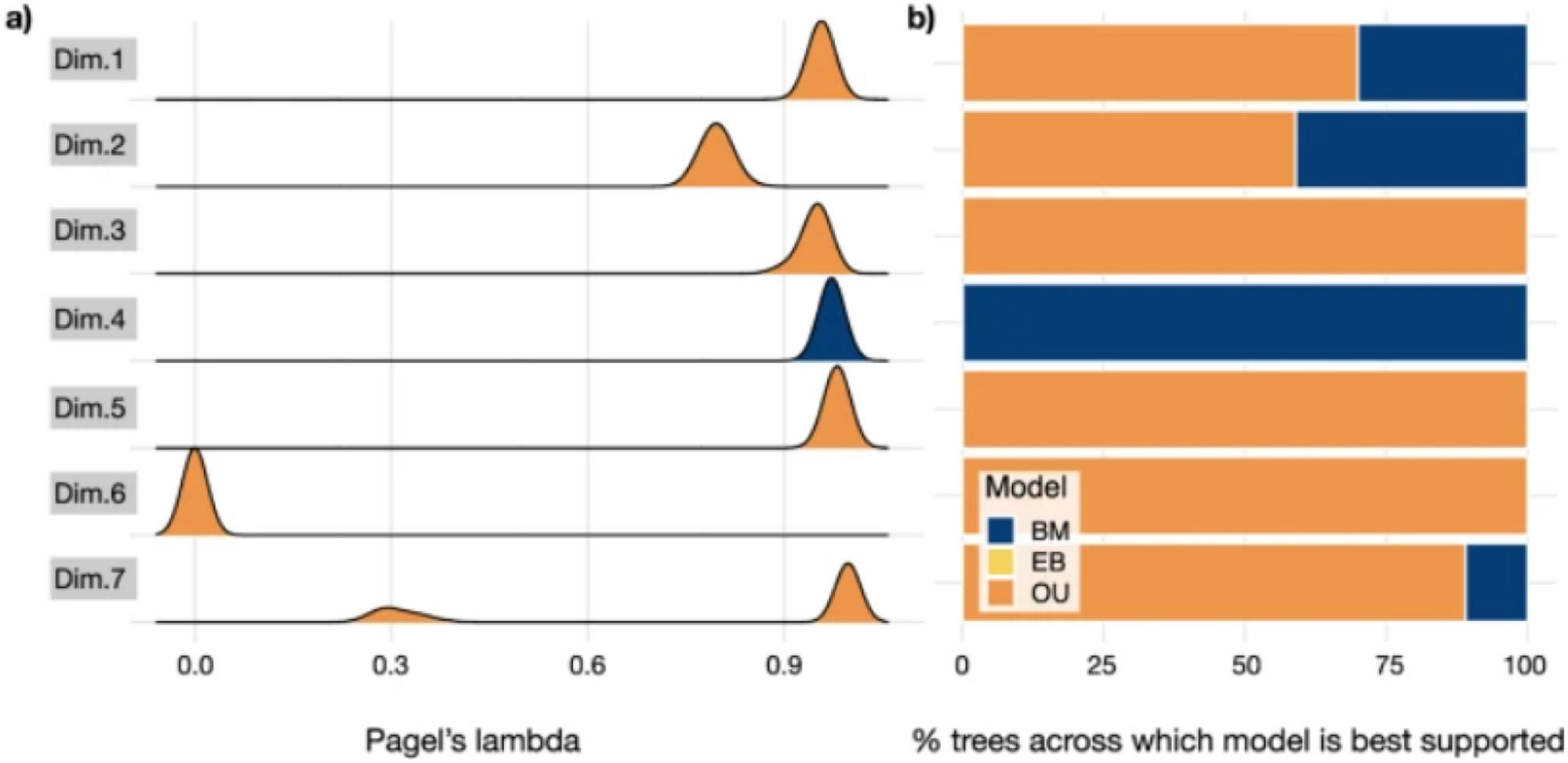# Publisher Correction: Multidimensional primate niche space sheds light on interspecific competition in primate evolution

**DOI:** 10.1038/s42003-024-06519-5

**Published:** 2024-07-05

**Authors:** L. A. van Holstein, H. D. McKay, C. Pimiento, K. Koops

**Affiliations:** 1 Clare College, University of Cambridge, Cambridge, UK; 2 Ape Behaviour & Ecology Group, Department of Evolutionary Anthropology, University of Zurich, Zurich, Switzerland; 3 Department of Archaeology, University of Cambridge, Cambridge, UK; 4 Department of Paleontology, University of Zurich, Zurich, Switzerland; 5 Department of Biosciences, Swansea University, Swansea, UK

**Keywords:** Evolution, Evolutionary ecology

Correction to: *Communications Biology* 10.1038/s42003-024-06324-0, published online 27 May 2024

In the original version of the Article, Figure 3 was incorrectly pasted in twice in the pdf version. This has now been corrected.

Original Figure 3:
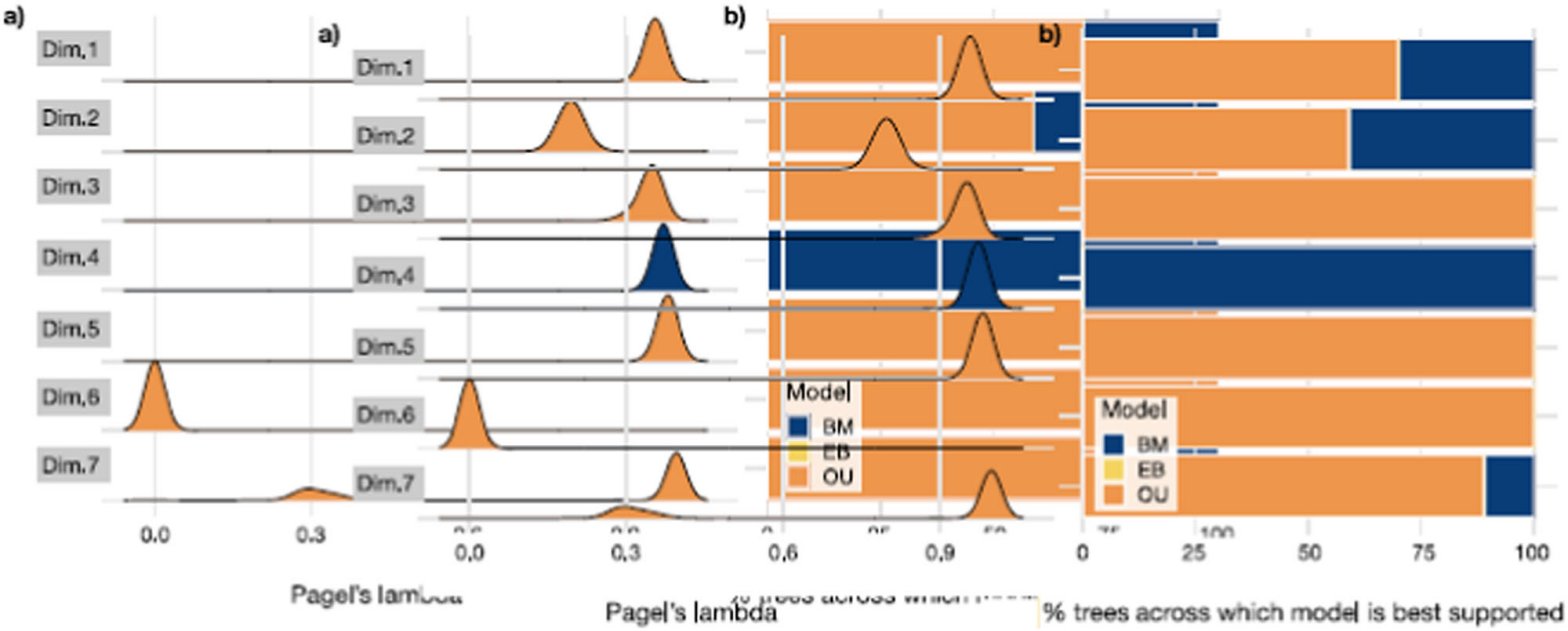


Corrected Figure 3: