# Supplementary material for: Multidimensional primate niche space sheds light on interspecific competition in primate evolution
Source: Commun Biol. 2024 May 27;7:647. doi: 10.1038/s42003-024-06324-0 (PMC11130132; doi:10.1038/s42003-024-06324-0)

SUPPLEMENTARY MATERIAL – FILE 1

Supplementary Table 1: Definition of variables extracted from databases

Variable	Database	Definition
**Body mass**	COMBINE	Mass of adult (or age unspecified) live or freshly-killed specimens (excluding pregnant females) using captive, wild, provisioned, or unspecified populations
**Life history**	Generated in analyses (see definition)	PC1 of PCA incorporating all life history variables
Longevity	COMBINE	Maximum reported age at death for the species in days
Female maturity	COMBINE	The amount of time needed for a female to reach sexual maturity in days
Gestation length	COMBINE	Length of time of fetal growth in days
Interbirth interval	COMBINE	Time between reproduction events in days
Weaning age	COMBINE	Age at which primary nutritional dependency on the mother ends<br>and independent foraging begins in days
Generation length	COMBINE	Average age of parents of the current cohort in days
**Diet breadth**	COMBINE	Number of dietary categories (with each category comprising more than 20% of a species' diet)
**Trophic guild**	Ecological Traits of the World's Primates	Assignment to one of six trophic guild categories
Folivory–frugivory	Ecological Traits of the World's Primates	Diet comprised of both fruits/seeds and leaves in similar proportions
Frugivory	Ecological Traits of the World's Primates	>60% of fruits/seeds in diet
Gummivory	Ecological Traits of the World's Primates	Diet dominated by plant exudates
Insectivory	Ecological Traits of the World's Primates	>50% of arthropods in diet
Omnivory	Ecological Traits of the World's Primates	Diet comprised of both plants and animals in similar proportions
Folivory	Ecological Traits of the World's Primates	>60% leaves in diet
**Foraging stratum**	COMBINE	Assignment to one of five foraging stratum categories
Ground foraging	COMBINE	Foraging on ground (Nowak 1999)
Scansorial foraging	COMBINE	Foraging between ground and canopy (Nowak 1999)
Arboreal foraging	COMBINE	Foraging in canopy (Nowak 1999)
**Activity cycle**	COMBINE	Time of the day in which the species carries out most of its activities
Cathemerality	COMBINE	Nocturnal/crepuscular, cathemeral, crepuscular or diurnal/crepuscular
Diurnality	COMBINE	Most activities carried out during the day
Nocturnal	COMBINE	Most activities carried out during the night

Supplementary Table 2: Eigenvalues for the dimensions of factor analysis of mixed data.

Seven dimensions were used in subsequent analyses.

	Eigenvalue	% of variance	Cumulative % of variance
Dim 1	2.65	15.58	15.58
Dim 2	2.05	12.04	27.62
Dim 3	1.79	10.54	38.16
Dim 4	1.61	9.50	47.65
Dim 5	1.23	7.26	54.91
Dim 6	1.12	6.61	61.52
Dim 7	1.02	6.00	67.52
Dim 8	0.99	5.82	73.34
Dim 9	0.89	5.22	78.57
Dim 10	0.76	4.48	83.04
Dim 11	0.68	4.01	87.05
Dim 12	0.52	3.04	90.09
Dim 13	0.45	2.67	92.76
Dim 14	0.42	2.46	95.21
Dim 15	0.39	2.27	97.48
Dim 16	0.24	1.41	98.89
Dim 17	0.19	1.11	100.00

Supplementary Figure 1: Scree plot of percentage of variance explained by each dimension of the multidimensional niche space

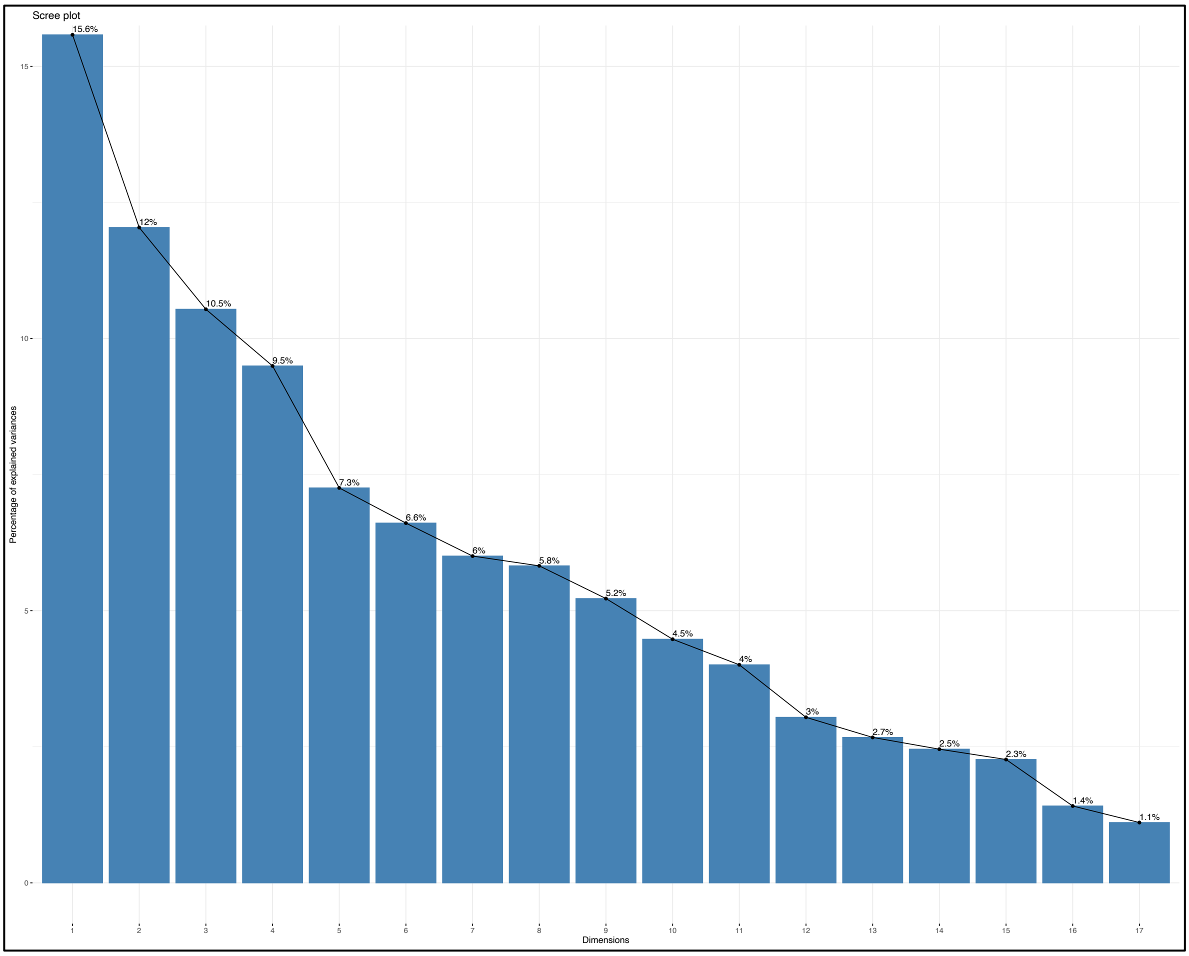

Supplementary Figure 2: Contributions of traits to dimensions 1-7 of the multidimensional niche space

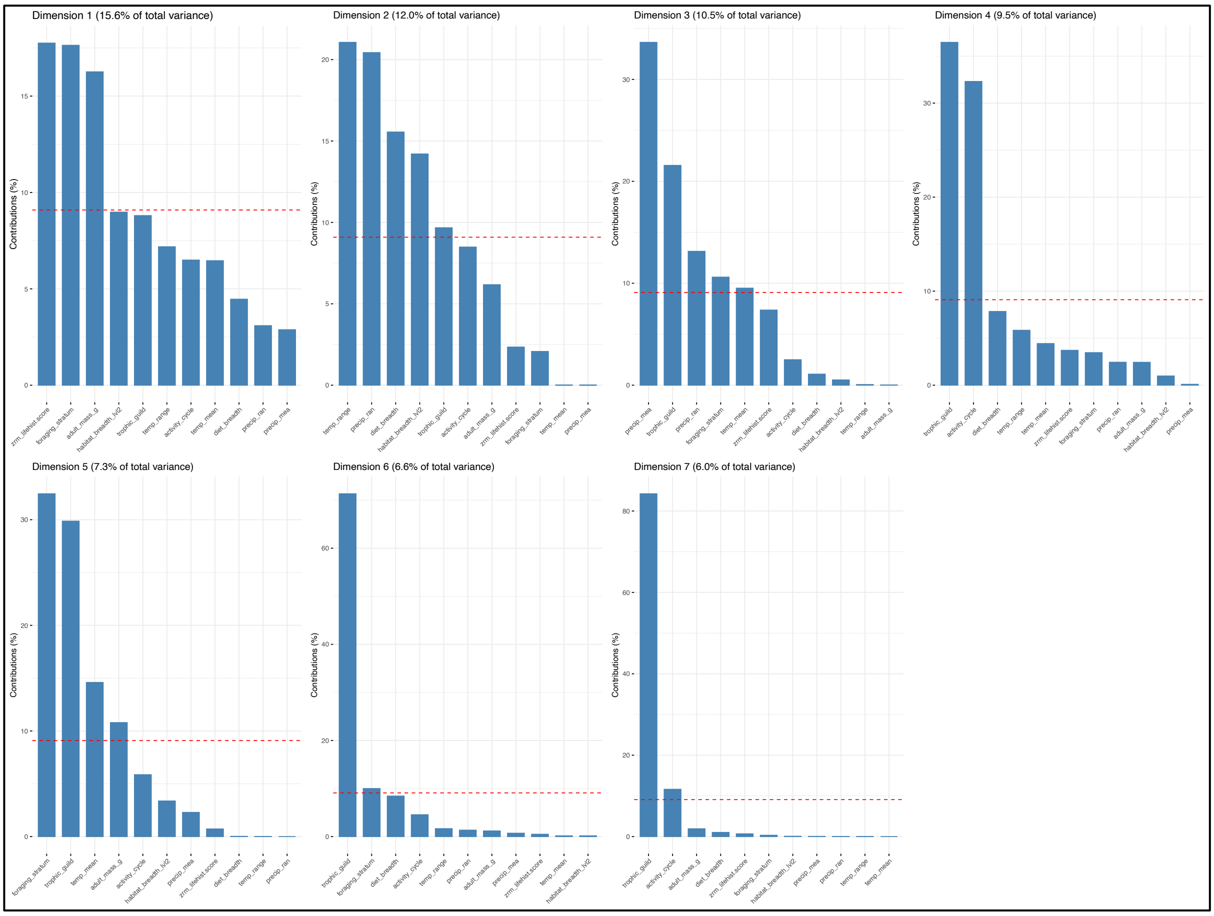

Supplementary Figure 3: The first three niche dimensions – occupation at highest level of taxonomy

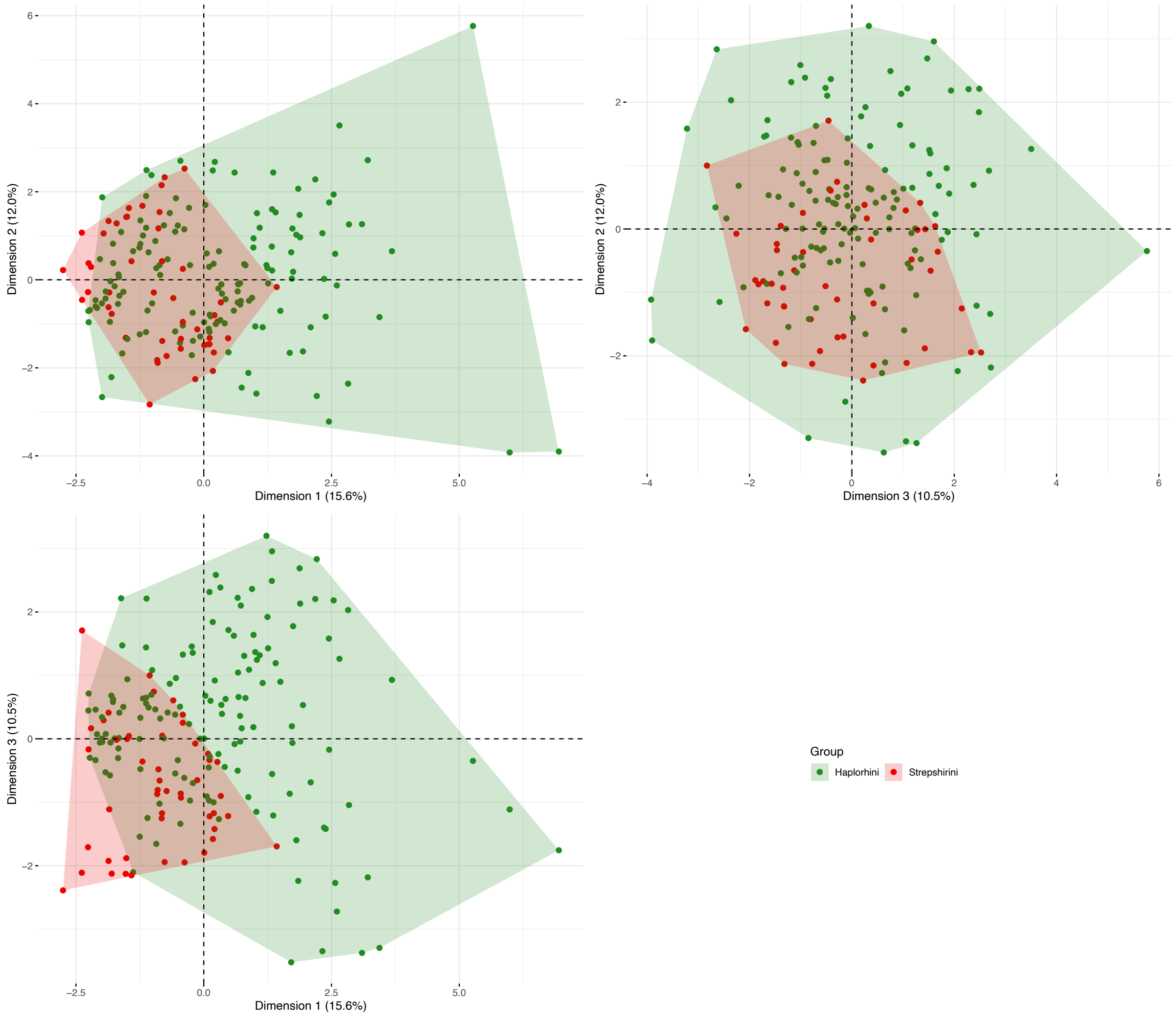

Supplementary Figure 4: The first three niche dimensions – occupation at intermediate level of taxonomy

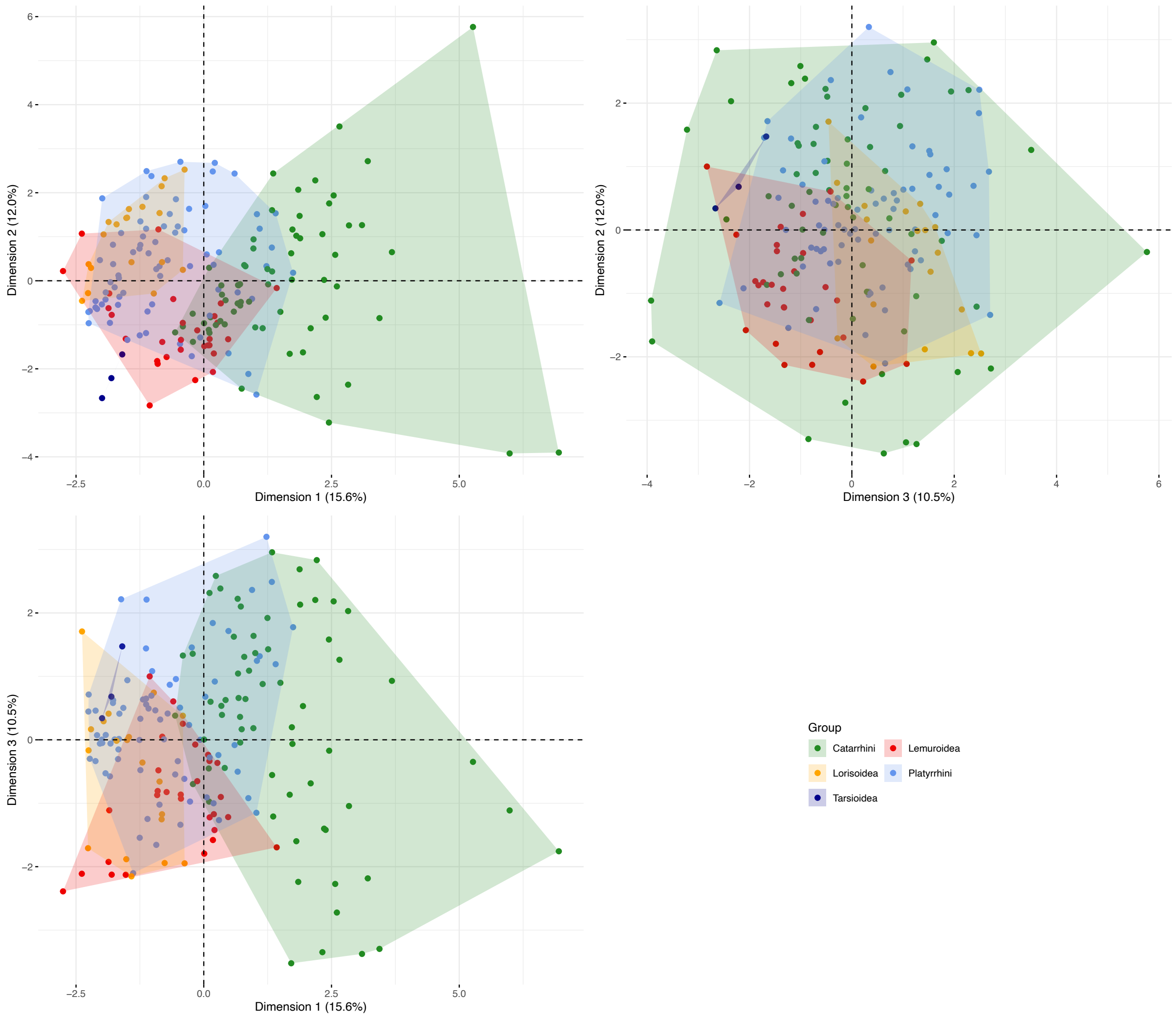

Supplementary Figure 5: Outliers for uniqueness and specialisation scores

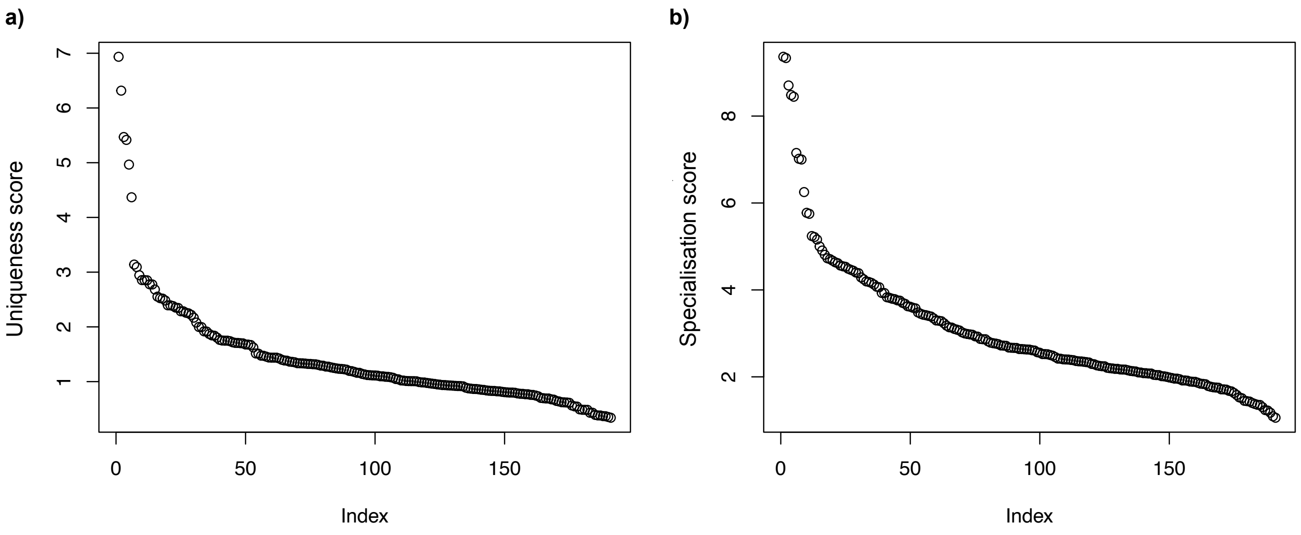

Supplementary Figure 6: **Correlation plot of quantitative niche variables.**

a) Correlation between variables before highly correlated life history variables were reduced to a single variable; b) Correlations between variables after highly correlated life history variables were reduced to a single variable

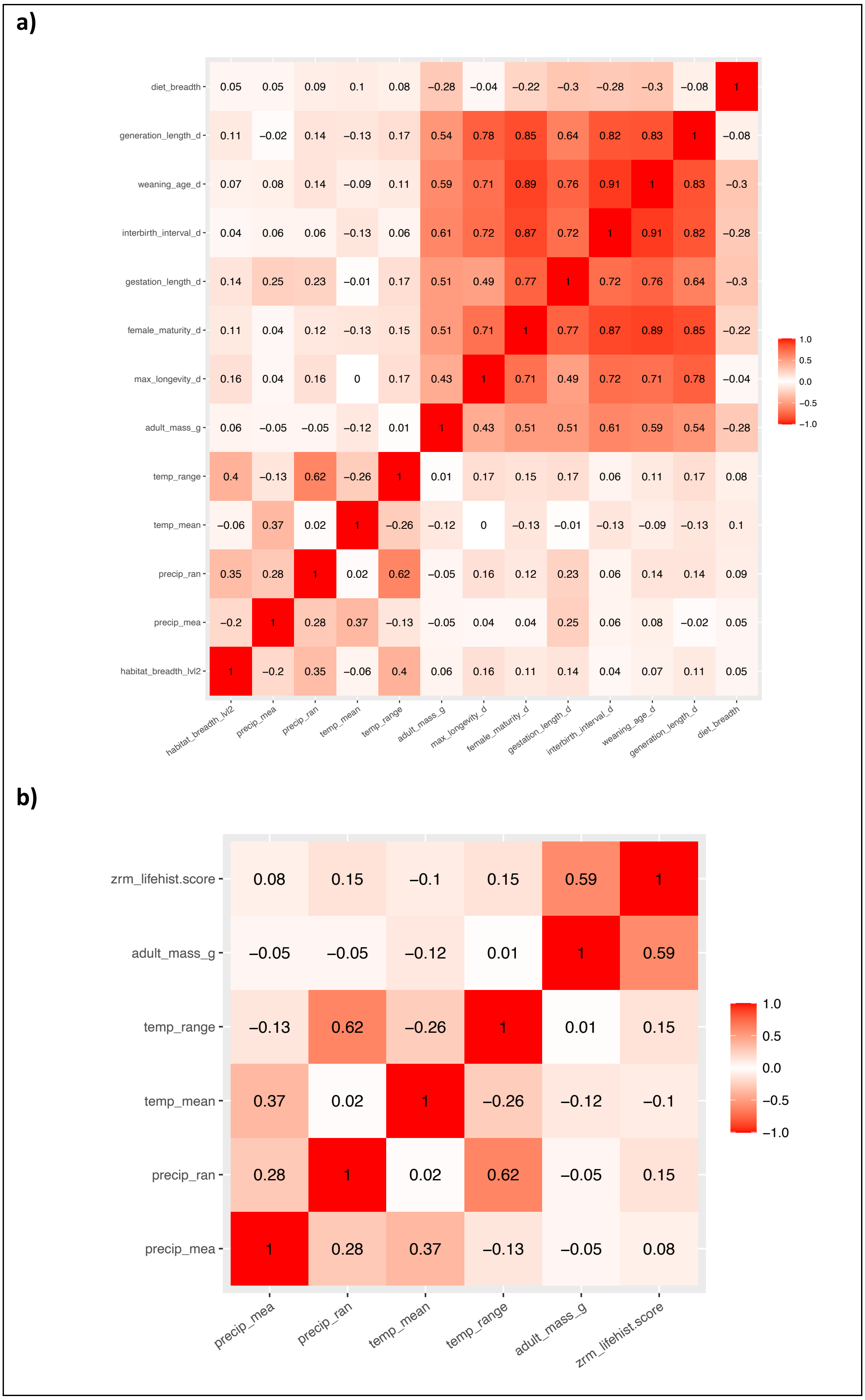

Supplement: Supplementary file 2 — Supplementary Information [file 42003_2024_6324_MOESM2_ESM.pdf]
