# Supplementary material for: Multidimensional primate niche space sheds light on interspecific competition in primate evolution
Source: Commun Biol. 2024 May 27;7:647. doi: 10.1038/s42003-024-06324-0 (PMC11130132; doi:10.1038/s42003-024-06324-0)
Supplement: Supplementary file 3 — Description of Additional Supplementary Materials [file 42003_2024_6324_MOESM3_ESM.docx]

**Description of Additional Supplementary Files**

**File name:** Supplementary Data 1

**Description:** Niche uniqueness and specialization scores for 191 primate species
