# Supplementary material for: Multidimensional primate niche space sheds light on interspecific competition in primate evolution
Source: Commun Biol. 2024 May 27;7:647. doi: 10.1038/s42003-024-06324-0 (PMC11130132; doi:10.1038/s42003-024-06324-0)
Supplement: Supplementary file 5 — Reporting summary [file 42003_2024_6324_MOESM5_ESM.pdf]

nature portfolio

Corresponding author(s): L A van Holstein

Last updated by author(s): 12 March 2024

Reporting Summary

Nature Portfolio wishes to improve the reproducibility of the work that we publish. This form provides structure for consistency and transparency in reporting. For further information on Nature Portfolio policies, see our Editorial Policies and the Editorial Policy Checklist.

Statistics

For all statistical analyses, confirm that the following items are present in the figure legend, table legend, main text, or Methods section.

n/a	Confirmed	
☐	☒	The exact sample size (*n*) for each experimental group/condition, given as a discrete number and unit of measurement
☒	☐	A statement on whether measurements were taken from distinct samples or whether the same sample was measured repeatedly
☐	☒	The statistical test(s) used AND whether they are one- or two-sided<br>*Only common tests should be described solely by name; describe more complex techniques in the Methods section.*
☐	☒	A description of all covariates tested
☐	☒	A description of any assumptions or corrections, such as tests of normality and adjustment for multiple comparisons
☒	☐	A full description of the statistical parameters including central tendency (e.g. means) or other basic estimates (e.g. regression coefficient) AND variation (e.g. standard deviation) or associated estimates of uncertainty (e.g. confidence intervals)
☐	☒	For null hypothesis testing, the test statistic (e.g. *F*, *t*, *r*) with confidence intervals, effect sizes, degrees of freedom and *P* value noted<br>*Give P values as exact values whenever suitable.*
☒	☐	For Bayesian analysis, information on the choice of priors and Markov chain Monte Carlo settings
☒	☐	For hierarchical and complex designs, identification of the appropriate level for tests and full reporting of outcomes
☒	☐	Estimates of effect sizes (e.g. Cohen's *d*, Pearson's *r*), indicating how they were calculated

Our web collection on statistics for biologists contains articles on many of the points above.

Software and code

Policy information about availability of computer code

Data collection

We obtained data relating to primate niche elements including life history, morphology, behaviour, ecology and climate. We extracted the following variables from the COMBINE database: six life history traits measured in days (longevity, female maturity, gestation length, interbirth interval, weaning age and generation length); adult body mass (in grams); foraging stratum (ground level, scansorial or arboreal); activity cycle (nocturnal, diurnal, or cathemeral); and diet breadth (number of dietary categories, comprising more than 20% of a species' diet). Activity cycle classifications were cross-checked and updated with current IUCN Red List of Threatened species (https://www.iucnredlist.org; last accessed 2 November 2023). We collected each species' trophic guild (folivore, frugivore, folivore and frugivore, omnivore, gummivore or insectivore) from the Ecological Traits of the World's Primates database, and any gaps were filled using IUCN Red List data and mammalDIET. Table S1 provides definitions of variables extracted from published databases. Habitat breadth, defined as the number of IUCN Red List level 2 habitat types deemed suitable for a given species, was collected from the IUCN Red List.We obtained climatic niche data using spatial analysis methods in QGIS Version 3.1430. We used bioclimatic data from WorldClim version 2 at a spatial resolution of 2.5 minutes. We took primate geographical ranges from the IUCN Red List spatial data and extracted the mean and range of both temperature and precipitation, as well as elevation range, within each species' geographical range, using the 'zonal statistics' tool in QGIS. Species with any missing or imputed data were removed from analyses. The final dataset consisted of values for 16 niche traits for 191 species.

Data analysis

i) Modelling Primate niches
All analyses were carried out in the R environment32. To prepare the trait data for Factor Analysis of Mixed Data (FAMD, see below), we first examined the correlations between traits (Figure S1a). The six life history variables were highly correlated (Pearson's r > 0.6), other than gestation length and maximum longevity (Pearson's r = 0.49). Highly correlated variables are likely to dominate the contribution to the principal components33, so we carried out a Principal Component Analysis (PCA) on the six life history variables. Principal component 1 of this analysis explained 80.6% of the variance in life history across species. We added the coordinates of the first principal component to the niche trait dataset, to represent the life history component of a niche in one metric. In this principal component – referred to hereafter as 'life

history score'. We removed the six original life history variables from the dataset, leaving eleven niche elements.

Using FAMD function of the 'FactoMineR' package 34, we then carried out factor analysis of mixed data on the niche elements. This method can use both categorial and continuous data to describe the variation in a dataset, potentially in a lower-dimension space. Additionally, this function automatically standardises the quantitative variables before analysis. When using categorical variables in this type of analysis, the same trait can contribute to several dimensions due to the multiple categories held within it35. We defined primate niche space as the space described by all dimensions with eigenvalues greater than one to comply with the Kaiser criterion (Table S2). Primate niches, then, refer to the location of a species within this multidimensional space.

ii) Quantifying niche uniqueness and specialisation
We followed the definitions put forward by Mouillot et al. (2013), Leitão et al. (2016) and Pimiento et al. (2020a), where uniqueness measures the isolation of a point relative to its nearest neighbours in multidimensional space, and ecological specialisation measures a point's isolation relative to the centre of the space. We measured the mean Euclidean distance of each species' niche location to its five nearest neighbours in multidimensional niche space to produce a niche uniqueness score for each species (i.e, the higher the score, the greater the mean distance from its nearest neighbours). Niche uniqueness, then, is a proxy for isolation of the focal species' location in niche space, and thus represents the inverse of redundancy (that is, the species does not have similar trait combinations to many others). To check that niche uniqueness scores were robust to the number of neighbours used in the calculation, we repeated the distance measurements with varying numbers of neighbours. The uniqueness score using the five nearest neighbours is strongly correlated with that using one ($\rho = 0.85$), three ($\rho = 0.99$), ten ($\rho = 0.97$) and 100 ($\rho = 0.79$) neighbours. Importantly, the top eight unique species – though their ranks differ slightly – are the same for five, ten, and 100 neighbours. Therefore, the measurement is robust to the number of neighbours used. The niche specialisation score was taken as the Euclidean distance of a species to the centroid of the multidimensionsional niche space24,36–38. Niche specialisation scores, then, capture how generalist (i.e., average combinations of niche elements, close to the centre of the space), or specialised (i.e., extreme combination of niche elements, far from the centre of the space) species are in niche space.

Niche uniqueness captures a species' relative isolation in niche space. It can therefore be used as a measure of intensity of interspecific competition, with species scoring highly likely not subject to strong competition from other primate species, and low scores indicating a focal species' niche is similar to those of other species. We report the top 10 highest and lowest-scoring species for niche uniqueness. This logic also means that niche factors positively contributing to niche uniqueness scores are those primates likely use to avoid niche competition.

To assess which factors of a niche predict niche uniqueness and specialisation, we ran the following PGLS regression across the same 100 primate phylogenies:
S=intercept+〖var〗_1+〖var〗_2+〖...+var〗_n
where S is specialisation or uniqueness score, scaled to have a range between zero and one, and var is niche variable (i.e., body mass, diet breadth, trophic guild, foraging stratum, activity cycle, habitat breadth, precipitation mean, precipitation range, temperature mean, temperature range, and life history). Continuous variables were centred and scaled to have unit variance. For each primate phylogeny, the p-values associated with each niche variable were adjusted for multiple testing using the Holm–Bonferroni method. We then counted the number of significant adjusted p-values across all 100 phylogenies for each niche variable.

iii) Exploring evolutionary dynamics: how do species' individual locations across individual niche dimensions evolve?
We first estimated phylogenetic signal of species' locations each niche dimension across 100 randomly selected primate node-dated DNA-only phylogenies from VertLife39. Discrepancies between the taxonomies utilized by the IUCN Red List and VertLife were resolved using the IUCN Red List synonyms. We therefore estimated the phylogenetic signal of primate niches using Pagel's [illegible]. We used the phylosig function in the 'phytools' package40. Likelihood ratio tests were used to evaluate if niche distribution across the primates was significantly different than expected in a Brownian motion (BM) model ([illegible] = 1) or a null model (no phylogenetic signal: [illegible] = 0). Reported p-values are Holm–Bonferroni corrected.

We then fit three maximum likelihood models of the evolution of species' locations across each niche dimension using the fit_t_standard function in the package 'RPANDA'41 across the 100 randomly selected primate phylogenies:

Brownian motion: ϕ(t)=σ_0^2
where t is time and σ is the 'rate' of evolution of the species' location (whether across a single dimension, or its niche uniqueness/ specialisation score);

Ornstein-Uhlenbeck (OU): dX_t=α([illegible]-X_t)+σdB_t
where t is time, Xt is the species' location (whether across a single dimension, or its niche uniqueness/specialisation score) at time t, σ is the 'rate' of evolution of a species' location, α is the strength of selection, and [illegible] is the adaptive optimum species' location;

Early burst: ϕ(t)=σ_0^2×e^(-rt)
where t is time and σ is the 'rate' of evolution of a species' location (whether across a single dimension, or its niche uniqueness/specialisation score), which declines through time as a function of rate r.

Model fits were compared using the small-sample corrected Akaike Information Criterion (AICc) weights, with the model with the lowest AICc score being selected as the best fit.

For manuscripts utilizing custom algorithms or software that are central to the research but not yet described in published literature, software must be made available to editors and reviewers. We strongly encourage code deposition in a community repository (e.g. GitHub). See the Nature Portfolio guidelines for submitting code & software for further information.

Data

Policy information about availability of data

All manuscripts must include a data availability statement. This statement should provide the following information, where applicable:

- Accession codes, unique identifiers, or web links for publicly available datasets
- A description of any restrictions on data availability
- For clinical datasets or third party data, please ensure that the statement adheres to our policy

All data will be made available on FigShare upon publication

Research involving human participants, their data, or biological material

Policy information about studies with human participants or human data. See also policy information about sex, gender (identity/presentation), and sexual orientation and race, ethnicity and racism.

Reporting on sex and gender	N/A
Reporting on race, ethnicity, or other socially relevant groupings	N/A
Population characteristics	N/A
Recruitment	N/A
Ethics oversight	N/A

Note that full information on the approval of the study protocol must also be provided in the manuscript.

Field-specific reporting

Please select the one below that is the best fit for your research. If you are not sure, read the appropriate sections before making your selection.

☐ Life sciences ☐ Behavioural & social sciences ☒ Ecological, evolutionary & environmental sciences

For a reference copy of the document with all sections, see nature.com/documents/nr-reporting-summary-flat.pdf

Ecological, evolutionary & environmental sciences study design

All studies must disclose on these points even when the disclosure is negative.

Study description	We describe and analyse seven-dimensional niche space, comprising 11 traits, for 191 primate species.
Research sample	191 primate species; data taken from online databases.
Sampling strategy	N/A
Data collection	We obtained data relating to primate niche elements including life history, morphology, behaviour, ecology and climate. We extracted the following variables from the COMBINE database: six life history traits measured in days (longevity, female maturity, gestation length, interbirth interval, weaning age and generation length); adult body mass (in grams); foraging stratum (ground level, scansorial or arboreal); activity cycle (nocturnal, diurnal, or cathemeral); and diet breadth (number of dietary categories, comprising more than 20% of a species' diet). Activity cycle classifications were cross-checked and updated with current IUCN Red List of Threatened species (https://www.iucnredlist.org; last accessed 2 November 2023). We collected each species' trophic guild (folivore, frugivore, folivore and frugivore, omnivore, gummivore or insectivore) from the Ecological Traits of the World's Primates database, and any gaps were filled using IUCN Red List data and mammalDIET. Table S1 provides definitions of variables extracted from published databases. Habitat breadth, defined as the number of IUCN Red List level 2 habitat types deemed suitable for a given species, was collected from the IUCN Red List.We obtained climatic niche data using spatial analysis methods in QGIS Version 3.1430. We used bioclimatic data from WorldClim version 2 at a spatial resolution of 2.5 minutes. We took primate geographical ranges from the IUCN Red List spatial data and extracted the mean and range of both temperature and precipitation, as well as elevation range, within each species' geographical range, using the 'zonal statistics' tool in QGIS. Species with any missing or imputed data were removed from analyses. The final dataset consisted of values for 16 niche traits for 191 species.
Timing and spatial scale	N/A
Data exclusions	Any species for which there was imputed data.

Reproducibility | N/A
Randomization | N/A
Blinding | N/A

Did the study involve field work? ☐ Yes ☒ No

Reporting for specific materials, systems and methods

We require information from authors about some types of materials, experimental systems and methods used in many studies. Here, indicate whether each material, system or method listed is relevant to your study. If you are not sure if a list item applies to your research, read the appropriate section before selecting a response.

Materials & experimental systems

n/a	Involved in the study
☒	☐ Antibodies
☒	☐ Eukaryotic cell lines
☒	☐ Palaeontology and archaeology
☒	☐ Animals and other organisms
☒	☐ Clinical data
☒	☐ Dual use research of concern
☒	☐ Plants

Methods

n/a	Involved in the study
☒	☐ ChIP-seq
☒	☐ Flow cytometry
☒	☐ MRI-based neuroimaging

Plants

Seed stocks | *Report on the source of all seed stocks or other plant material used. If applicable, state the seed stock centre and catalogue number. If plant specimens were collected from the field, describe the collection location, date and sampling procedures.*

Novel plant genotypes | *Describe the methods by which all novel plant genotypes were produced. This includes those generated by transgenic approaches, gene editing, chemical/radiation-based mutagenesis and hybridization. For transgenic lines, describe the transformation method, the number of independent lines analyzed and the generation upon which experiments were performed. For gene-edited lines, describe the editor used, the endogenous sequence targeted for editing, the targeting guide RNA sequence (if applicable) and how the editor was applied.*

Authentication | *Describe any authentication procedures for each seed stock used or novel genotype generated. Describe any experiments used to assess the effect of a mutation and, where applicable, how potential secondary effects (e.g. second site T-DNA insertions, mosiacism, off-target gene editing) were examined.*
