# Supplementary material for: Multidimensional primate niche space sheds light on interspecific competition in primate evolution
Source: Commun Biol. 2024 May 27;7:647. doi: 10.1038/s42003-024-06324-0 (PMC11130132; doi:10.1038/s42003-024-06324-0)
Supplement: Supplementary file 1 — Peer Review File [file 42003_2024_6324_MOESM1_ESM.pdf]

nature portfolio

Peer Review File

Multidimensional primate niche space sheds light on interspecific competition in primate evolution

Open Access This file is licensed under a Creative Commons Attribution 4.0 International License, which permits use, sharing, adaptation, distribution and reproduction in any medium or format, as long as you give appropriate credit to the original author(s) and the source, provide a link to the Creative Commons license, and indicate if changes were made. In the cases where the authors are anonymous, such as is the case for the reports of anonymous peer reviewers, author attribution should be to 'Anonymous Referee' followed by a clear attribution to the source work. The images or other third party material in this file are included in the article's Creative Commons license, unless indicated otherwise in a credit line to the material. If material is not included in the article's Creative Commons license and your intended use is not permitted by statutory regulation or exceeds the permitted use, you will need to obtain permission directly from the copyright holder. To view a copy of this license, visit http://creativecommons.org/licenses/by/4.0/.

REVIEWERS' COMMENTS:

Reviewer #1 (Remarks to the Author):

Although the manuscript could be more concise, in the revised version the authors have done an excellent work to address and incorporate all the feedback. The results are interesting and well supported by the models. I'm happy to recommend the paper for publication.
